# COVID-19 severity is related to poor executive function in people with post-COVID conditions

**DOI:** 10.1007/s00415-023-11587-4

**Published:** 2023-03-20

**Authors:** Mar Ariza, Neus Cano, Bàrbara Segura, Ana Adan, Núria Bargalló, Xavier Caldú, Anna Campabadal, Maria Angeles Jurado, Maria Mataró, Roser Pueyo, Roser Sala-Llonch, Cristian Barrué, Javier Bejar, Claudio Ulises Cortés, NAUTILUS Project Collaborative Group, Jose A. Bernia, Vanesa Arauzo, Marta Balague-Marmaña, Berta Valles-Pauls, Jesús Caballero, Anna Carnes-Vendrell, Gerard Piñol-Ripoll, Ester Gonzalez-Aguado, Carme Tayó-Juli, Eva Forcadell-Ferreres, Silvia Reverte-Vilarroya, Susanna Forné, Jordina Muñoz-Padros, Anna Bartes-Plan, Jose A. Muñoz-Moreno, Anna Prats-Paris, Inmaculada Rico, Nuria Sabé, Laura Casas, Marta Almeria, Maria José Ciudad, Anna Ferré, Manuela Lozano, Tamar Garzon, Marta Cullell, Sonia Vega, Sílvia Alsina, Maria J. Maldonado-Belmonte, Susana Vazquez-Rivera, Sandra Navarro, Eva Baillès, Maite Garolera, Carme Junqué

**Affiliations:** 1 Unitat de Psicologia Mèdica, Departament de Medicina, Universitat de Barcelona, Barcelona, Spain; 2 Institut de Neurociències, Universitat de Barcelona, Barcelona, Spain; 3 Grup de Recerca en Cervell, Cognició I Conducta, Consorci Sanitari de Terrassa (CST), Terrassa, Spain; 4 Institut d’Investigacions Biomèdiques August Pi I Sunyer (IDIBAPS), Barcelona, Spain; 5 Centro de Investigación Biomédica en Red Sobre Enfermedades Neurodegenerativas (CIBERNED), Barcelona, Spain; 6 Departament de Psicologia Clínica I Psicobiologia, Universitat de Barcelona, Barcelona, Spain; 7 Diagnostic Imaging Centre, Hospital Clínic de Barcelona, Universitat de Barcelona, Barcelona, Spain; 8 Centro de Investigación Biomédica en Red de Salud Mental (CIBERSAM), Instituto de Salud Carlos III, Barcelona, Spain; 9 Institut de Recerca de Sant Joan de Déu (IRSJD), Esplugues de Llobregat, Barcelona, Spain; 10 Departament de Biomedicina, Universitat de Barcelona, Barcelona, Spain; 11 Centro de Investigación Biomédica en Red en Bioingeniería, Biomateriales Y Nanomedicina (CIBER-BBN), Barcelona, Spain; 12 Departament de Ciències de La Computació, Universitat Politècnica de Catalunya-BarcelonaTech, Barcelona, Spain; 13 Neuropsychology Unit, Consorci Sanitari de Terrassa (CST), Terrassa, Spain; 14 Departament de Ciències Bàsiques, Universitat Internacional de Catalunya, Sant Cugat del Vallès, Spain

**Keywords:** COVID-19, Executive function, Neuropsychological test, Post-acute COVID-19 syndrome, Symptom assessment

## Abstract

Patients with post-coronavirus disease 2019 (COVID-19) conditions typically experience cognitive problems. Some studies have linked COVID-19 severity with long-term cognitive damage, while others did not observe such associations. This discrepancy can be attributed to methodological and sample variations. We aimed to clarify the relationship between COVID-19 severity and long-term cognitive outcomes and determine whether the initial symptomatology can predict long-term cognitive problems. Cognitive evaluations were performed on 109 healthy controls and 319 post-COVID individuals categorized into three groups according to the WHO clinical progression scale: severe-critical (*n* = 77), moderate-hospitalized (*n* = 73), and outpatients (*n* = 169). Principal component analysis was used to identify factors associated with symptoms in the acute-phase and cognitive domains. Analyses of variance and regression linear models were used to study intergroup differences and the relationship between initial symptomatology and long-term cognitive problems. The severe-critical group performed significantly worse than the control group in general cognition (Montreal Cognitive Assessment), executive function (Digit symbol, Trail Making Test B, phonetic fluency), and social cognition (Reading the Mind in the Eyes test). Five components of symptoms emerged from the principal component analysis: the “Neurologic/Pain/Dermatologic” “Digestive/Headache”, “Respiratory/Fever/Fatigue/Psychiatric” and “Smell/ Taste” components were predictors of Montreal Cognitive Assessment scores; the “Neurologic/Pain/Dermatologic” component predicted attention and working memory; the “Neurologic/Pain/Dermatologic” and “Respiratory/Fever/Fatigue/Psychiatric” components predicted verbal memory, and the “Respiratory/Fever/Fatigue/Psychiatric,” “Neurologic/Pain/Dermatologic,” and “Digestive/Headache” components predicted executive function. Patients with severe COVID-19 exhibited persistent deficits in executive function. Several initial symptoms were predictors of long-term sequelae, indicating the role of systemic inflammation and neuroinflammation in the acute-phase symptoms of COVID-19.” *Study Registration*: www.ClinicalTrials.gov, identifier NCT05307549 and NCT05307575.

**Supplementary Information:**

The online version supplementary material available at 10.1007/s00415-023-11587-4.

## Introduction

The post-coronavirus disease 2019 (COVID-19) condition (PCC) manifests 3 months after the onset of the disease, and presents with symptoms that remain for at least 2 months and cannot be explained by other diseases [1]. PCC is characterized by a wide variety of fixed or fluctuating symptoms, including cognitive complaints. While 60%-80% of patients with PCC report experiencing brain fog, memory, loss of attentional focus, and language disturbances [2–4], objective evaluations of people with PCC have shown impairments in attention, processing speed, memory, and executive functions [5–7].

The severity of COVID-19 and post-COVID cognitive impairment assessed through systematic neuropsychological assessments was first shown to be related in hospitalized patients with acute disease [8]. Intensive care unit (ICU) stay has been linked to reduced executive function, and the need for oxygen therapy has been linked to reduced performance in several cognitive measures 10–40 days after hospital discharge. Over the medium-long term, the general severity of acute illness has been related to residual cognitive deficits [9], treatment required for respiratory symptoms has been related to worse global cognitive performance [10], respiratory distress to lower processing speed [11], and hypoxemia to impaired long-term memory and visuospatial learning at five months but not at the one-year evolution [12].

Additional evidence has been obtained from studies comparing hospitalized and non-hospitalized patients. In comparison with non-hospitalized patients, hospitalized individuals are more likely to have impairments in attention, executive functioning, category fluency, and verbal memory [13] or slower processing speed [5]. Post-ICU patients showed a lower cognitive composite score than non-ICU patients. However, among non-ICU patients, the cognitive composite score did not differ between those who were hospitalized and those who were not [14]. In a similar study performed with a healthy control (HC) group, patients with severe PCC showed lower processing speed than those with mild-moderate PCC and healthy control participants [15]. In a Finnish study, both ICU and hospitalized patients underperformed patients treated at home in the total cognitive score at 6 months post-COVID. Moreover, ICU participants underperformed hospitalized patients and HCs in the attention domain [16].

However, in multiple investigations using samples from 18 to 478 hospitalized and non-hospitalized participants with acute illnesses, the severity of COVID-19 was not associated with cognitive impairments at 3–4 months [17–19]. According to a recent meta-analysis, patients admitted to the hospital during the acute infection were less likely to report post-COVID cognitive symptoms than outpatients three months (or more) after the disease [20].

Another aspect that requires consideration is the predictive value of acute symptomatology for long-term cognitive impairment. Severe acute respiratory syndrome coronavirus 2 (SARS-CoV-2) can infect several human cell types, as seen by COVID-19's vast array of symptoms. Typical signs and symptoms include fever, fatigue, gastrointestinal issues, cough, sore throat, shortness of breath, myalgia, headaches, dizziness, and changes in smell and taste [21–23]. The etiology of cognitive dysfunction may originate from the pathophysiology of acute illness [24]. However, it is currently unknown whether the effects of COVID-19 on the brain are caused by virus invasion in the brain, oxygen deprivation of the brain, or the body's excessive inflammatory response in seriously affected individuals [25]. Acute symptoms, even if they are not neurological manifestations, could contribute to the understanding of post-COVID cognitive problems. In a split study, Guo et al. found that initial illness-related symptoms explained part of the variation in post-COVID subjective cognitive symptoms [3]. They then demonstrated how some aspects of neuropsychological performance can also be explained by acute sickness symptoms [26].

Despite the number of studies in the field, the relationship between cognitive outcomes and the severity of COVID-19 is still not completely clear, probably because the underlying mechanisms of the cognitive deficits identified are mostly unknown. This study aimed to clarify the relationship between the severity of COVID-19 and long-term cognitive outcomes in a large sample of participants, including a control group. Our second objective was to determine if the initial symptomatology can predict long-term cognitive impairment. Since COVID-19 symptoms are highly diverse and heterogeneous, we aimed to use principal component analysis to identify phenotypes or clinical symptoms that frequently coexist.

## Methods

### Participants

The NAUTILUS Project is a cross-sectional observational study of post-COVID-19 cognitive consequences based on multimodal data. We used the available clinical and neuropsychological data of the project in the present study. The sample consisted of 428 participants, including 319 participants with PCC and 109 HC individuals who were evaluated at the Neuropsychology and COVID Units of 16 Hospitals in Catalonia, Madrid, and Andorra, coordinated by the Consorci Sanitari de Terrassa (Terrassa, Barcelona, Spain). The inclusion criteria for the PCC group were as follows: (a) confirmed diagnosis of COVID-19 according to WHO criteria with signs and symptoms of the disease during the acute phase; (b) at least 12 weeks after infection; and (c) age over 18 years. Exclusion criteria were as follows: (a) established diagnosis of psychiatric, neurological, neurodevelopmental disorder, or systemic pathologies known to cause cognitive deficits before the episode of COVID-19, and (b) motor or sensory alterations that impeded neuropsychological examination. The HCs did not have COVID-19 (no positive test or compatible symptoms) and were selected after applying the same exclusion criteria as in the PCC group. All participants were native Spanish speakers.

### Procedure

The overall procedure consisted of two sessions. In the first session, various questionnaires were administered to collect information about demographic factors and behaviors related to the participants’ health and medical history. Participants with PCC were questioned about their COVID-19 experience and the symptoms they were experiencing at the time of evaluation. For a list of typical acute COVID-19 acute symptoms, presence/absence and the number of days were recorded. We developed a scale from 0 to 4, in which 0 indicated the absence of the symptom and 4 indicated a long-lasting symptom. Next, participants rated the severity of their COVID-19 experience on a visual analog scale of 1–10. Later, they were asked about the symptoms they were currently experiencing (post-COVID symptoms) and whether these were minor, major, or different from those experienced in the acute phase. Finally, we asked them to report any other symptoms they had been experiencing and had not been covered in the interview.

In the second session, each participant underwent a cognitive assessment with a comprehensive neuropsychological battery. We used the Montreal Cognitive Assessment (MoCA) for general cognitive screening [27, 28]. The WAIS-IV Digit Span subtest was used to measure verbal attention (digit span forward) and working memory (digit span backward) [29]. To assess verbal memory, we used the Spanish version of Rey's Auditory Verbal Learning Test (RAVLT) [30, 31]. Visual scanning, tracking, and motor speed were assessed by the Digit Symbol Coding Test (WAIS-III) [29]. Parts A and B of the Trail Making Test (TMT) were administered to measure visual scanning, motor speed and attention, and mental flexibility [32]. A difference score (B-A) that removed the speed element from the test evaluation was calculated [33]. The Controlled Oral Word Association Test (COWAT) [34, 35] was used to evaluate verbal fluency and language. The number of words beginning with the letters P, M, and R recalled in 1 min was recorded. Semantic fluency was evaluated using the category “animals” [36]. The number of correct animals reported in 1 min was counted. The interference score of the Stroop test was calculated as a measure of cognitive inhibitory control [37]. The Boston Naming Test (BNT) was used to evaluate language [38]. Social cognition was assessed with the Reading the Mind in the Eye Test (RMET) [39]. The Word Accentuation Test (TAP) was used to estimate the premorbid intelligence quotient (IQ) [40]. In addition to cognitive measures, we used the Chalder Fatigue Scale (CFQ) [41] to assess fatigue, the Generalized Anxiety Disorder 7-item scale (GAD-7) [42, 43] to assess anxiety, and the Patient Health Questionnaire-9 (PHQ-9) to assess depressive symptoms [44, 45]. The quality of life was evaluated by the WHOQOL-BREFF [46]. Trained neuropsychologists performed all evaluations.

The recruitment was conducted between June 2021 and June 2022. The study was conducted with the approval of the Drug Research Ethics Committee (CEIm) of Consorci Sanitari de Terrassa (CEIm code: 02–20-107–070) and the Ethics Committee of the University of Barcelona (IRB00003099). All participants provided written informed consent.

### Statistical analyses

Descriptive statistics were obtained for all variables of the study. Group differences in demographics were examined by conducting an analysis of variance (ANOVA). The Chi-square test was performed to compare binarized measures between the groups. One-way analysis of covariance (ANCOVA) with Bonferroni-adjusted post-hoc comparisons was performed to determine group differences in cognitive functioning. Graphical representations and descriptive statistics were used to study the assumptions. The effect size was calculated using the value partial eta squared (ή_p_^2^). To investigate if the cognitive symptoms of PCC were predicted by the acute-phase symptoms, principal component analysis (PCA) was performed first on 21 auto-reported acute-phase symptoms and on Z-scores of 15 neuropsychological variables to define the cognitive domains, followed by linear regressions (stepwise) with the acute symptom components as predictors and the neuropsychological components as dependent variables. Analyses were performed using IBM SPSS Statistics 27.0 (IBM Corporation, Armonk, NY, USA) and R Statistical Software (version 4.2.0; The R Foundation for Statistical Computing Platform). The critical level for statistical significance was set at α = 0.05.

## Results

### Sample demographics

The 319 participants with PCC were classified into three groups according to the WHO clinical progression scale [47]: severe-intensive care unit (ICU-PCC) (*n* = 77), hospitalized (H-PCC) (*n* = 73), and mild (M-PCC) (*n* = 169) (Table 1). The participants’ sociodemographic characteristics and comorbidities are shown in Table 2. The M-PCC and the HC groups were equivalent in age and sex, had a higher proportion of women, and were younger than the ICU-PCC and the H-PCC groups. The three PCC groups showed no differences in formal education and estimated IQ. However, the education level and estimated IQ in the HC group were higher than those in all three PCC groups. Thus, age, sex, educational level, and estimated IQ were covariates in comparing cognitive results among the four groups. On average, all PCC participants had shown a positive test 320 days before their neuropsychological evaluation (SD = 156.66 days), and the ICU-PCC group had fewer days of evolution since the start of COVID-19 than the other two groups. Premorbid high blood pressure and obesity were more prevalent among ICU participants than the other PCC and HC groups.

**Table 1:** Clinical characteristics of the PCC groups based on the WHO clinical progression scale

	WHO clinical progression scale score	N (%)
ICU-PCC	6–9	77 (24%)
*IMV*		*38 (49.4%)*
* NIV or HFNC*		*39 (50.6%)*
H-PCC	4–5	73 (23%)
*NIV or HFNC*		*25 (34.2%)*
*Mask or nasal prongs*		*37 (50.7%)*
*No O*_2_* treatment*		*11 (15.1%)*
M-PCC	2–3	169 (53%)
*Disturbance of ADL*		*139 (82.3%)*
*No disturbance in ADL*		*30 (17.7%)*

*PCC* post-COVID condition, *ICU* intensive care unit, *H* hospitalized, *M* mild, *IMV* invasive mechanical ventilation, *NIV* non-invasive ventilation, *HFNC* high-flow nasal cannula, *ADL* activities of daily living

**Table 2:** Sociodemographic characteristics and comorbidities of the PCC severity and HC groups

	ICU-PCC *n* = 77	H-PCC *n* = 73	M-PCC *n* = 169	HC *n* = 109			
	Mean (SD)	Mean (SD)	Mean (SD)	Mean (SD)	F	p	Post-hoc
Age	51.91 (8.32)	52.69 (7.39)	46.21 (9.23)	46.10 (9.31)	15.71	0.0001	ICU > HC ICU > M H > HC H > M
Education (years)	13.14 (3.19)	13.34 (3.50)	14.26 (3.28)	15.57 (2.93)	11.07	0.0001	HC > ICU HC > H HC > M
Estimated IQ^*^	100.75 (7.82)	101.46 (8.25)	101.85 (7.73)	104.79 (6.58)	5.43	0.001	HC > ICU HC > H HC > M
Time of evolution^**^	269.75 (104.10)	303.51 (131.49)	350.56 (178.87)		7.842	0.0001	M > ICU
	**N (%)**	**N (%)**	**N (%)**	**N (%)**	**χ**^**2**^	**p**	
Sex (% female)	34 (44.2%)	35 (50%)	130 (77%)	84 (77.1%)	41.98	0.0001	
***Comorbidities***							
Heart disease	4 (5.2%)	3 (4.1%)	4 (2.4%)	3 (2.8%)			
Respiratory disease	11 (14.3%)	10 (13.7%)	19 (11.2%)	5 (4.6%)	10.00	0.124	
Chronic kidney disease	1 (1.3%)	1 (1.4%)	1 (0.6%)	0			
High blood pressure	22 (28.6%)	13 (17.8%)	12 (7.1%)	5 (4.6%)	33.61	0.0001	
Dyslipidemia	16 (20.8%)	13 (17.8%)	17 (10.1%)	11 (10.1%)	9.40	0.152	
Diabetes mellitus	5 (6.5%)	7 (9.6%)	1 (0.6%)	3 (2.8%)			
Obesity	41 (53.2%)	26 (35.6%)	32 (18.9%)	16 (14.7%)	46.30	0.0001	
Chronic liver disease	4 (5.2%)	5 (6.8%)	1 (0.6%)	0			
Tobacco smoking	3 (3.9%)	2 (2.7%)	17 (10.1%)	27 (24.8%)			

*PCC* = post-COVID condition, *ICU* intensive care unit, *H* hospitalized, *M* mild, *HC* healthy control, *IQ* intelligence quotient

*Intelligence estimated by means of Word Accentuation Test

**Time of evolution is the days since first positive test

#### Differences in cognitive performance

Table 3 shows the fatigue, depression, anxiety, and quality of life scores for each PCC severity and HC group. The CFQ, PHQ-9, GAD-7, and WHOQOL-BREF scores were significantly different among groups. Post-hoc analysis showed that the CFQ, PHQ-9, and GAD-7 scores were higher in the PCC than in the HC group. Individuals in the M-PCC group had higher fatigue and depression levels than those in the H-PCC group. The quality of life assessed by the WHOQOL-BREFF was better in the HC group than in the PCC groups. We used fatigue, depression, and anxiety as covariates in the cognitive analysis. However, we also analyzed the data without these mood and fatigue variables (Supplementary Table 1).

**Table 3:** Intergroup differences in fatigue, mood, and quality of life measures adjusted for age, sex, educational level, and estimated IQ*

	ICU-PCC (*n* = 77)	H-PCC (*n* = 73)	M-PCC (*n* = 169)	HC (*n* = 109)					
	M_adj_ (SE)	M_adj_ (SE)	M_adj_ (SE)	M_adj_ (SE)	F	p	η^2^	Post-hoc Bonferroni	p
CFQ score	5.88 (0.49)	5.23 (0.50)	6.68 (0.32)	1.80 (0.41)	31.207	0.0001	0.190	ICU > HC H > HC M > HC M > H	0.0001 0.0001 0.0001 0.017
PHQ-9 score	8.64 (0.70)	7.35 (0.72)	9.88 (0.45)	3.35 (0.58)	27.42	0.0001	0.172	ICU > HC H > HC M > HC M > H	0.0001 0.0001 0.0001 0.004
GAD-7 score	7.61 (0.60)	6.15 (0.61)	6.49 (0.39)	3.34 (0.50)	11.773	0.0001	0.082	ICU > HC H > HC M > HC	0.0001 0.0001 0.0001
WHOQOL-BREF score	58.14 (12.38)	58.23 (13.82)	56.59 (13.19)	67.20 (9.86)	16.650	0.0001	0.110	ICU < HC H < HC M < HC	0.0001 0.0001 0.0001

*PCC* post-COVID condition, *ICU* intensive care unit, *H* = hospitalized, *M* mild, *HC* healthy control, *CFQ* Chandler Fatigue Scale, *PHQ-9* Patient Health Questionnaire-9, *GAD-7* Generalized Anxiety Disorder 7-item scale, *WHOQOL-BREF* World Health Organization Quality of Life Scale (General quality of life)

*Adjusted by age, sex, educational level, and estimated IQ

η^2^ effect size is as follows: η^2^ = 0.009, small; η^2^ = 0.059, medium; η^2^ = 0.139, large

**Fig. 1:**
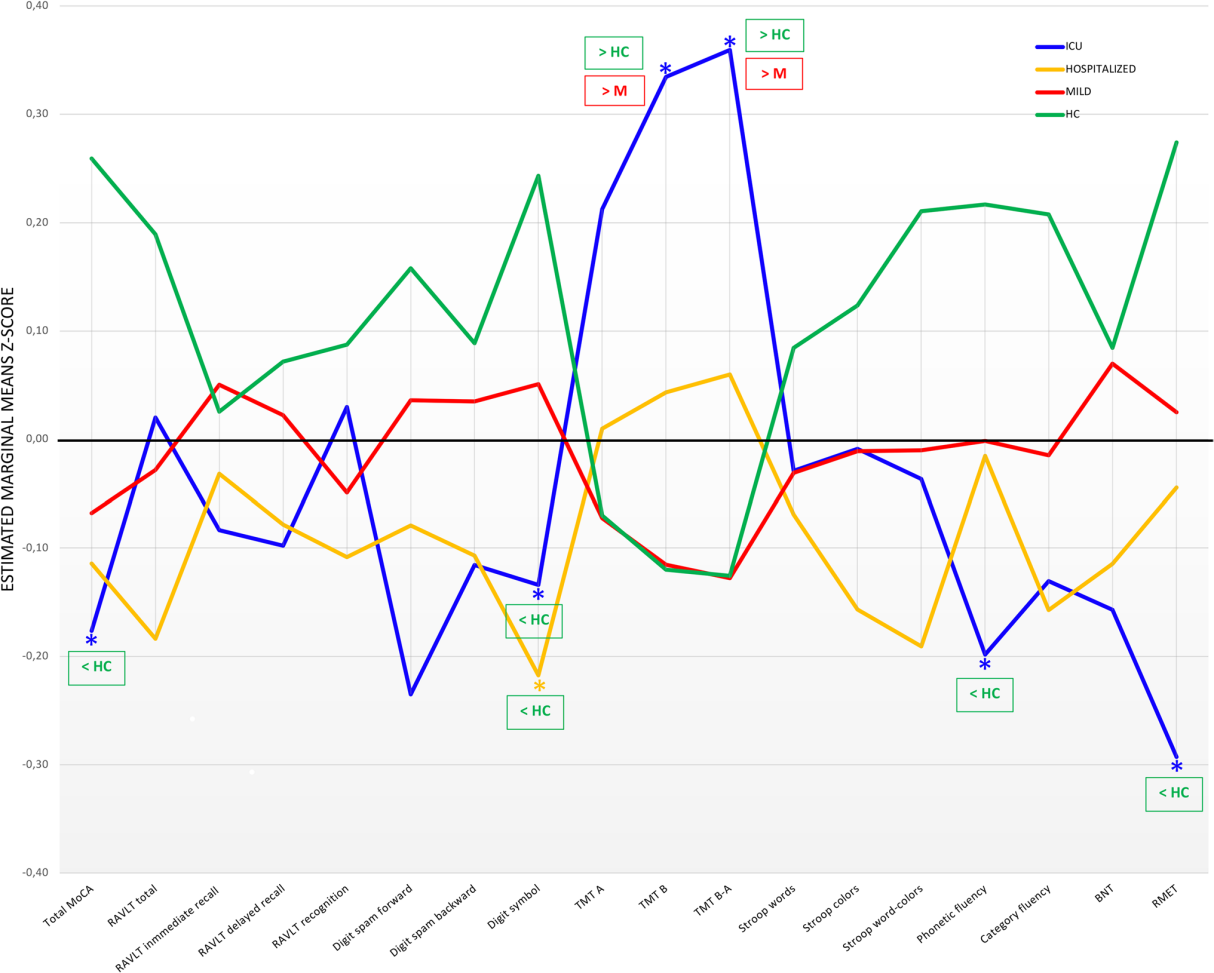
Cognitive profiles of the post-COVID condition severity groups and healthy controls. Healthy controls (HCs) are presented in green, ICU-PCC in blue, H-PCC in yellow, and M-PCC in red. Data are presented as means of Z-scores adjusted by age, sex, educational level, estimated IQ, fatigue, depression, and anxiety test scores. Lower Z-scores indicate poorer performance, except for TMT (time), where lower Z-scores indicate better performance. Statistically significant differences between groups are marked with an asterisk

The groups showed statistically significant differences in MoCA, Digit symbol, TMT-B, TMT-B-A, phonetic fluency, and the RMET scores after controlling for age, sex, educational level, estimated IQ, fatigue, depression, and anxiety test scores. The ICU-PCC group performed worse in the MoCA, Digit symbol, TMT B, TMT-B-A, phonetic fluency, and RMET assessments than the HC group and obtained poorer results than the M-PCC group in the TMT-B and TMT-B-A assessments. The H-PCC group showed worse performance in the Digit symbol assessments than the HC group (Table 4 and Fig. 1).

**Table 4:** Adjusted* means of the neuropsychological variables in the PCC severity and HC groups

	ICU-PCC (*n* = 77)	H-PCC (*n* = 73)	M-PCC (*n* = 169)	HC (*n* = 109)					
	M_adj_ (SE)	M_adj_ (SE)	M_adj_ (SE)	M_adj_ (SE)	F	p	η_p_^2^	Post-hoc Bonferroni	p
MoCA score	25.91 (0.30)	26.08 (0.31)	26.21 (0.20)	27.13 (0.27)	3.606	0.014	0.027	ICU < HC	0.021
RAVLT total score	45.28 (0.98)	43.35 (1.00)	44.82 (0.66)	46.87 (0.88)	2.366	0.071	0.018		
RAVLT immediate recall score	8.76 (0.32)	8.92 (0.33)	9.17 (0.22)	9.09 (0.29)	0.381	0.767	0.003		
RAVLT delayed recall score	8.63 (0.36)	8.74 (0.36)	9.07 (0.24)	9.23 (0.32)	0.581	0.628	0.004		
RAVLT recognition score	12.41 (0.29)	12.06 (0.29)	12.21 (0.19)	12.55 (0.26)	0.665	0.574	0.005		
Digit span forward score	5.38 (0.14)	5.57 (0.14)	5.71 (0.10)	5.56 (0.12)	2.109	0.099	0.016		
Digit span backward score	4.33 (0.14)	4.34 (0.14)	4.51 (0.10)	4.58 (0.13)	0.773	0.509	0.006		
Digit symbol score	63.81 (1.98)	62.17 (2.00)	67.44 (1.34)	71.22 (1.78)	4.176	0.006	0.031	ICU < HC H < HC	0.047 0.006
TMT-A (time) score	41.39 (2.49)	37.09 (2.52)	35.34 (1.68)	35.39 (2.23)	1.440	0.231	0.011		
TMT-B (time) score	103.92 (6.32)	86.98 (6.44)	77.73 (4.24)	77.47 (5.62)	4.268	0.006	0.032	ICU > HC ICU > M	0.017 0.005
TMT-B-A (time) score	63.78 (4.80)	50.53 (4.89)	42.23 (3.23)	42.33 (4.27)	4.972	0.002	0.037	ICU > HC ICU > M	0.009 0.002
Stroop word score	94.20 (2.56)	93.33 (2.56)	94.16 (1.71)	96.66 (2.27)	0.353	0.787	0.003		
Stroop color score	65.34 (1.61)	65.22 (1.61)	65.31 (1.07)	67.23 (1.43)	1.102	0.348	0.008		
Stroop interference score	39.16 (1.22)	37.38 (1.22)	39.46 (0.81)	42.01 (1.10)	2.606	0.051	0.020		
Phonetic fluency (PMR) score	40.14 (1.38)	42.60 (1.40)	42.79 (0.93)	45.73 (1.24)	2.816	0.039	0.021	ICU < HC	0.024
Semantic fluency (animals) score	20.73 (0.62)	20.58 (0.63)	21.41 (0.42)	22.71 (0.59)	2.469	0.062	0.019		
BNT score	51.46 (0.59)	51.69 (0.60)	52.68 (0.40)	52.76 (0.53)	1.410	0.239	0.011		
RMET score	21.37 (0.45)	22.37 (0.45)	22.65 (0.30)	23.64 (0.40)	4.448	0.004	0.033	ICU < HC	0.002

*PCC* post-COVID condition, *ICU* intensive care unit, *H* hospitalized, *M* mild, *HC* healthy control, *MoCA* Montreal Cognitive Assessment, *RAVLT* Rey’s auditory verbal Learning Test, *TMT* Trail Making Test, *BNT* Boston Naming Test, *RMET* Reading the Mind in the Eyes Test

*Adjusted by age, sex, educational level, estimated IQ, Chalder Fatigue Scale (CFQ) score, Generalized Anxiety Disorder 7-item scale (GAD-7) score, and Patient Health Questionnaire-9 (PHQ-9) score

η_p_^2^ effect size is as follows: η_p_^2^ = 0.009, small; η_p_^2^ = .0.059, medium; η_p_^2^ = .0.139, large

Table 5 shows the frequency of acute-phase symptoms for each severity group and all the PCC participants. ICU stay was associated with greater limb weakness and the presentation of delirium and psychotic symptoms. Hospitalization was associated with fever. A higher proportion of PCC participants at home had headache, muscle and joint pain, changes in smell and taste, nasal congestion, and sore throat. The three groups did not show differences in the perception of COVID-19 severity measured with the visual analog scale (ICU: mean = 7.91, SD = 2.22; H: mean = 7.86, SD = 1.65; M: mean = 7.05, SD = 2.41).

**Table 5:** Reported signs and symptoms in the acute infection period in the PCC severity groups

	ICU-PCC *n* = 77	H-PCC *n* = 73	M-PCC *n* = 169			Total *n* = 319
	N (%)	N (%)	N (%)	χ^2^	p	N (%)
Tiredness	65 (84.4%)	68 (93.2%)	153 (92.7%)	4.971	0.083	286 (90.8%)
Fever	66 (85.7%)	68 (93.2%)	121 (72.9%)	15.01	0.0001	254 (80.6%)
Headache	44 (57.1%)	50 (68.5%)	144 (86.7%)	27.174	0.0001	237 (75.2%)
Muscle and joint pain	48 (62.3%)	46 (63%)	137 (82.5%)	15.820	0.0001	229 (72.7%)
Breathing issues	55 (71.4%)	51 (69.9%)	97 (58.8%)	4.879	0.087	204 (64.8%)
Cough	41 (53.2%)	48 (65.8%)	112 (67.5%)	4.786	0.091	200 (63.5%)
Loss of appetite	40 (51.9%)	46 (63%)	101 (60.8%)	2.301	0.316	187 (59.4)
Loss of smell	25 (32.5%)	30 (41.1%)	114 (67.5%)	31.360	0.0001	169 (54.2%)
Loss of taste	27 (35.1%)	30 (41.1%)	106 (62.7%)	19.982	0.0001	163 (52.2%)
Shaking chills	35 (45.5%)	37 (50.7%)	100 (60.2%)	5.174	0.075	171 (54.3%)
Limb weakness	52 (67.5%)	34 (46.6%)	72 (43.6%)	12.480	0.002	158 (50.3%)
Paresthesia	25 (32.5%)	27 (37%)	65 (38.5)	1.009	0.604	117 (36.7%)
Dizziness	27 (35.1%)	32 (43.8%)	86 (51.8%)	7.244	0.124	145 (46%)
Nasal congestion	28 (36.4%)	28 (38.4%)	86 (51.1%)	7.000	0.030	141 (40.4%)
Chest pain	30 (39%)	29 (39.7%)	84 (50.6%)	4.048	0.132	142 (45.1%)
Sore throat	22 (28.6%)	20 (27.4%)	93 (56%)	25.310	0.0001	134 (42.5%)
Diarrhea	24 (31.2%)	28 (38.4%)	76 (45.8%)	4.844	0.089	128 (40.1%)
Nausea	20 (26%)	25 (34.2%)	52 (31.3%)	1.271	0.530	97 (30.8%)
Conjunctival congestion	11 (14.3%)	15 (20.5%)	40 (24.1%)	3.071	0.215	66 (21.0%)
Skin rash/Discoloration of fingers or toes	9 (11.7%)	9 (12.3%)	33 (16.1%)	3.626	0.163	51 (16.2%)
Tachycardia	6 (7.8%)	7 (9.6%)	17 (10.1%)	0.323	0.851	30 (9.4%)
Seizures	1 (1.3%)	1 (1.4%)	0			2 (0.6%)
Stroke	0	2 (2.7%)	1 (0.6%)			2 (0.6%)
Menstrual cycle issues*	1 (12.5%)	0	8 (12.7%)			9 (11.5%)
Depression	37 (48.1%)	39 (53.4%)	79 (47.6%)	0.731	0.694	155 (49.2%)
Anxiety	27 (35.1%)	32 (43.8%)	77 (46.4%)	2.774	0.250	136 (43.2%)
Psychotic symptoms	24 (31.2%)	8 (11%)	4 (2.4%)	43.116	0.0001	36 (11.4%)
Delirium	30 (39%)	1 (1.4%)	0			32 (10%)
Obsessive–compulsive symptoms	5 (6.5%)	4 (5.5%)	15 (9%)			24 (7.6%)

*PCC* post-COVID condition, *ICU* intensive care unit, *H* hospitalized, *M* mild, *HC* healthy control

*% women under 45 years (*n* = 78)

#### Effect of acute symptoms on long-term cognition

PCA with initial symptoms was performed with a varimax orthogonal rotation to facilitate interpretability. The Kaiser–Meyer–Olkin (KMO) value (0.834) and Bartlett's test of sphericity (χ^2^_(210)_ = 1571.92; *p* < 0.000) indicated that the data were likely factorizable. PCA revealed five components with eigenvalues more significant than one, which explained 24.92%, 8.17%, 6.56%, 5.71%, and 5.11% of the total variance and were classified as “Digestive/Headache” (nausea, loss of appetite, dizziness, diarrhea, shaking chills, and headache), “Respiratory/Fever/Fatigue/Psychiatric” (depressive symptoms, anxious symptoms, psychotic symptoms, breathing issues, fever, and fatigue), “Neurologic/Pain/Dermatologic” (paresthesia, skin problems, limb weakness, and muscle and joint pain), “Smell/Taste” (smell and taste symptoms), and “Cold” (nasal and conjunctival congestion and cough), respectively. The factor scores were computed through the regression method. The rotated (varimax) component loadings for the initial symptoms are shown in Table 6.

**Table 6:** Factor and loading in PCA of symptoms

		Components			
**Symptom**	1	2	3	4	5
Nausea	**0.700**				0.314
Loss of appetite	**0.602**				
Dizziness	**0.599**				
Diarrhea	**0.566**				
Shaking chills	0.491				
Headache	0.445				
Depressive symptoms		**0.608**			
Anxiety symptoms		**0.605**			
Psychotic symptoms		**0.597**			
Shortness of breath		0.489			0.440
Fever		0.484			
Fatigue		0.436			
Skins symptoms			**0.699**		
Paresthesia			**0.699**		
Limb weakness			**0.507**		
Muscle and joint pain	0.377		0.463		
Smell alterations				**0.906**	
Taste alterations				**0.896**	
Nasal congestion					**0.720**
Conjunctival congestion			0.463		**0.638**
Cough	0.376				**0.524**

Component 1: *Digestive/Headache*: nausea, loss of appetite, dizziness, diarrhea, shaking chills, and headache

Component 2: *Respiratory/Fever/Fatigue/Psychiatric*: breathing issues, fever, depressive symptoms, anxious symptoms, psychotic symptoms, and fatigue

Component 3: *Neurologic/Pain/Dermatologic*: skins problems, limb weakness, paresthesia, and muscle and joint pain

Component: *Smell/Taste*: smell alterations, taste alterations

Component: *Cold*: nasal congestion, conjunctival congestion, cough

Bold indicates elements that charge above 0.5; the numbers that are not in bold are those that are loaded above 0.3

**Fig. 2:**
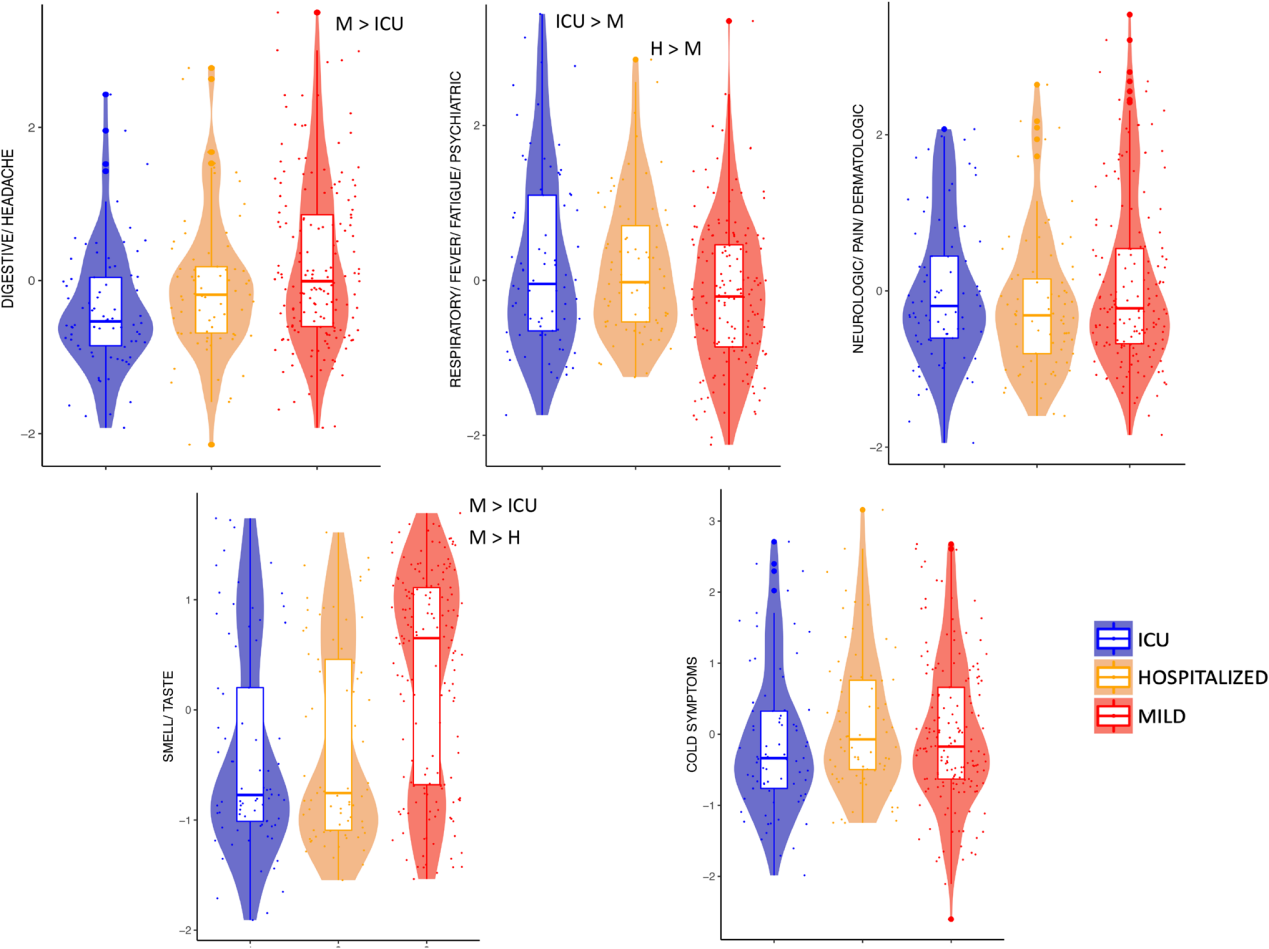
Violin plot for symptom factors across of PCC severity groups. Violin plots show the distribution for each symptom factor. Statistically significant differences were noted between PCC severity groups in Digestive/Headache, Respiratory/Fever/Fatigue/Psychiatric and the Smell/Taste score

The scores for the Digestive/Headache, Respiratory/Fever/Fatigue/Psychiatric, and Smell/Taste components were significantly different among the severity groups. Post-hoc analysis showed that the Digestive/Headache score was higher in the M-PCC group than in the ICU-PCC group; the Respiratory/Fever/Fatigue/Psychiatric score was higher in the ICU-PCC and H-PCC groups than in the M-PCC group, and the Smell/Taste score was higher in the M-PCC than in the ICU-PCC and H-PCC groups (Fig. 2 and Supplementary Table 2).

**Fig. 3:**
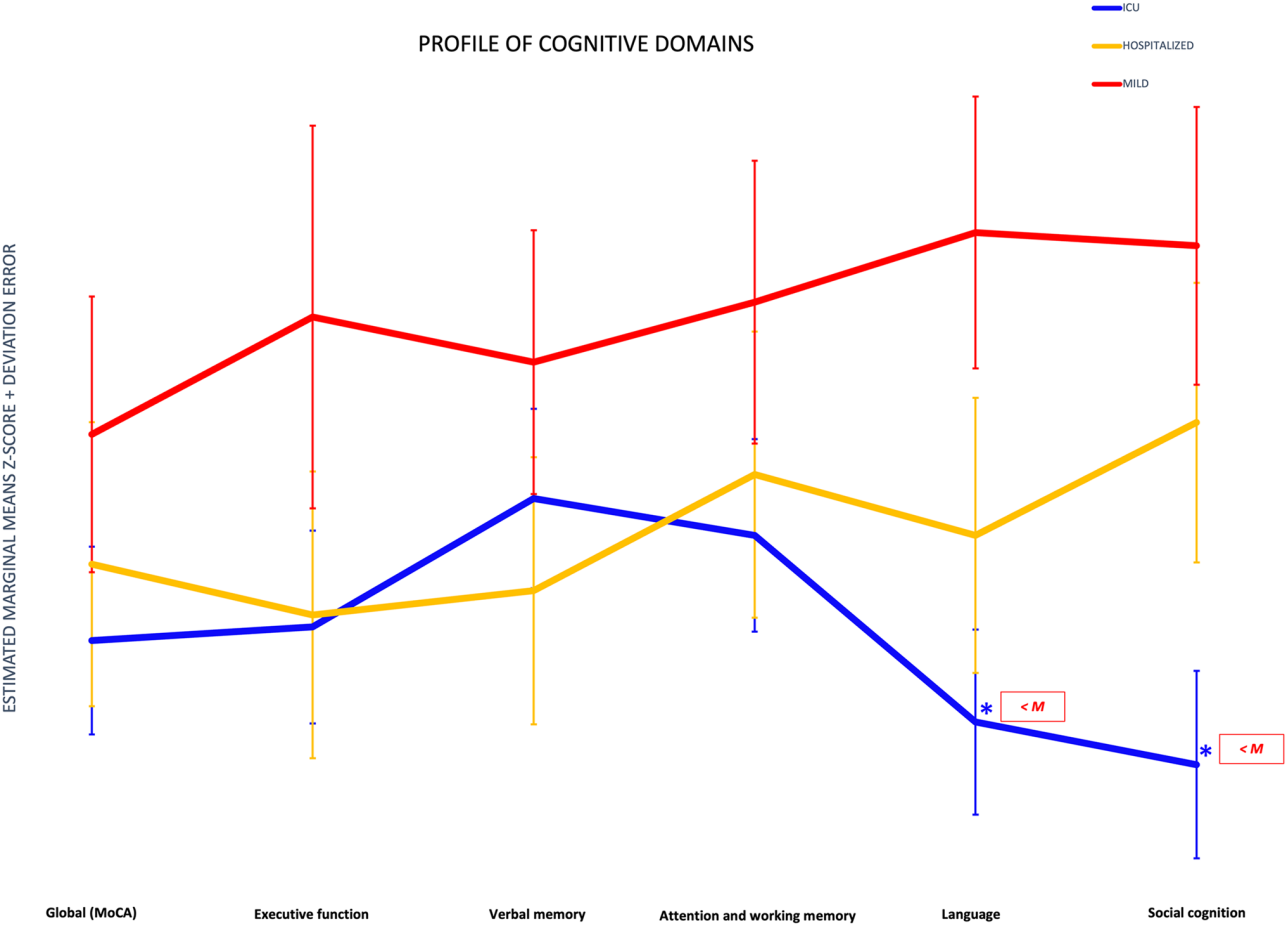
Cognitive domain profiles for the post-COVID conditions severity groups. ICU-PCC in blue, H-PCC in yellow, and M-PCC in red. Data are presented as means of Z-scores (adjusted by age, sex, educational level, time of evolution, fatigue, and depression test scores) and deviation error bars. Lower Z-scores indicate poorer performance. Statistically significant differences were noted between PCC severity groups (marked with an asterisk)

PCA with neuropsychological variables was performed with a direct oblimin rotation to facilitate interpretability. We excluded the scores obtained with the MoCA (a screening tool covering several cognitive domains) and the RMET (social cognition domain). All assumptions were met: overall KMO = 0.910 and Bartlett's test (χ^2^_(105)_ = 3878.99, *p* = 0.0001). PCA revealed four components as the best factorial solution, which explained 72.71% of the total variance (45.14%, 12.86%, 8.14%, and 6.57%). We classified the four components as the following cognitive domains: executive function (TMT, Symbol Digit, Stroop task), verbal memory (RAVLT), attention and working memory (WM) (Digits span forward and backward), and language (Phonetic fluency, Semantic fluency, BNT). The regression approach was used to calculate the factor scores. Component loadings of the rotated solution are presented in Table 7. Figure 3 shows the profile of the cognitive domains for the PCC severity and HC groups corrected for age, sex, educational level, time of evolution, fatigue, and depression test scores.

**Table 7:** Factor and loading in PCA of neuropsychological variables

		Component			
	1	2	3	4	
Troop words (Z score)	0.865				
Stroop colors (Z score)	0.828				
TMT-A (Z score)	− 0.786				
Stroop word-colors (Z score)	0.719				
TMT-B (Z score)	− 0.708				
Digit symbol (Z score)	0.640				
RAVLT delayed recall (Z score)		0.954			
RAVLT immediate recall (Z score)		0.950			
RAVLT learning (Z score)		0.865			
RAVLT recognition (Z score)		0.826			
Digit span backward (Z score)			0.896		
Digit span forward (Z score)			0.876		
BNT (Z score)				0.891	
Semantic fluency (animals) (Z score)				0.692	
Phonetic fluency (PMR) (Z score)				0.656	

Component 1: executive functions; Component 2: verbal memory; Component 3: attention and working memory (WM); Component 4: language

Linear regressions (stepwise) with the five acute symptom components as predictors and the neuropsychological components as dependent variables were performed. In addition to the four cognitive components, MoCA and RMET scores were used as dependent variables in multiple linear regression. The linear regression models were adjusted for potential confounders (age, sex, years of education, time of evolution, premorbid high blood pressure and obesity).

As seen in Table 8, the “Neurologic/Pain/Dermatologic”, “Digestive/Headache”, “Respiratory/Fever/Fatigue/Psychiatric” and “Smell/ Taste” components added statistical significance to the prediction of MoCA scores. Executive function was predicted by the “Respiratory/Fever/Fatigue/Psychiatric,” “Neurologic/Pain/Dermatologic,” and “Digestive/Headache” components. The “Neurologic/Pain/Dermatologic” and “Respiratory/Fever/Fatigue/Psychiatric” components added statistical significance to the prediction of verbal memory scores, and the attention and WM component was predicted by the “Neurologic/Pain/Dermatologic” component. The language and social cognition components were not explained by any acute-phase symptom component but by the variables for demographic characteristics and premorbid conditions.

**Table 8:** Multiple linear regression models testing the association between acute symptoms and cognitive performance

MoCA						
*F*	*p*	*R*^2^_adj_	Predictors	Beta	*t*	*p*
21.727	< 0.001	0.282	Constant		− 7.943	0.0001
			Years of education	0.387	7.447	0.0001
			Neurologic/Pain/Dermatologic factor	− 0.228	− 4.426	0.0001
			Digestive/ Headache factor	− 0.154	− 2.992	0.003
			Respiratory/Fever/Fatigue/Psychiatric factor	− 0.139	− 2.715	0.007
			Smell/ Taste	− 0.132	− 2. 577	0.010

**Executive Function component** (TMT, Stroop, Digit symbol)						
*F*	*p*	*R*^2^_adj_	Predictors	Beta	*t*	*p*
17.006	< 0.001	0.242	Constant		− 0.279	0.780
			Years of education	0.227	4.017	0.0001
			Respiratory/Fever/Fatigue/Psychiatric factor	− 0.233	− 4.346	0.0001
			Neurologic/ Pain/ Dermatologic factor	− 0.215	− 4.007	0.0001
			Digestive/ Headache factor	− 0.171	− 3.182	0.002
			Age	− 0.161	− 2.881	0.004

**Verbal memory component** (RAVLT)						
*F*	*p*	*R*^2^_adj_	Predictors	Beta	*t*	*p*
17.798	< 0.001	0.233	Constant		0.485	0.628
			Age	− 0.246	− 4.476	0.0001
			Years of education	0.197	3.639	0.0001
			Neurologic/ Pain/ Dermatologic factor	− 0.188	− 3.659	0.0001
			Respiratory/Fever/Fatigue/Psychiatric factor	− 0.152	− 2.943	0.004
			Sex (female)	0.130	2.453	0.015

**Language component** (Phonetic and semantic fluency, BNT)						
*F*	*p*	*R*^2^_adj_	Predictors	Beta	*t*	*p*
28.508	< 0.001	0.240	Constant		− 6.159	0.0001
			Years of education	0.232	8.780	0.0001
			Sex (male)	− 0.131	− 3.064	0.002
			Age	0.169	2.991	0.003

**Attention and WM component** (Digit span forward, Digit span backward)						
*F*	*p*	*R*^2^_adj_	Predictors	Beta	*t*	*p*
7.713	< 0.001	0.124	(Constant)		0.428	0.669
			Years of education	0.179	2.984	0.003
			Neurologic/ Pain/ Dermatologic factor	− 0.184	− 3.219	0.001
			Age	− 0.157	− 2.585	0.010
			Sex (male)	− 0.134	− 2.302	0.022

**Social cognition** (RMET)						
*F*	*p*	*R*^2^_adj_	Predictors	Beta	*t*	*p*
20.738	< 0.001	0.119	(Constant)		− 5.866	0.0001
			Years of education	0.330	5.960	0.0001
			Obesity	− 0.117	− 2.108	0.036

*MoCA* Montreal Cognitive Assessment, *TMT* Trail Making Test, *RAVLT* Rey’s auditory verbal Learning Test, *WM* working memory, *RMET* Reading the Mind in the Eyes Test

*Neurologic/Pain/Dermatologic component*: skin problems, limb weakness, paresthesia, and muscle and joint pain

*Respiratory/Fever/Fatigue/Psychiatric component*: breathing issues, fever, depressive symptoms, anxious symptoms, psychotic symptoms, and fatigue

*Digestive/Headache component*: nausea, loss of appetite, dizziness, diarrhea, shaking chills, and headache

*Smell/Taste component*: smell and taste alterations

## Discussion

The primary objective of the present study was to elucidate the link between COVID-19 severity and long-term cognitive outcomes. Previous studies have shown inconsistent results: some have reported a relationship [5, 8–16], while others did not identify any severity variable explaining cognitive performance [17–20]. Comparisons of these studies are challenging because their conclusions were drawn using various designs and methodologies. Moreover, only a few studies were specifically designed to examine this association [16, 18]. Some studies did not categorize patients according to the severity of their acute illness [8, 9, 11, 12, 19], or if they did, this categorization was only partially done or did not include a control group [5, 10, 13–15, 18]. Other studies only correlated the results of selected cognitive tests with severity assessments [10–12, 17]. Only one previous study compared groups according to the acute care environment and employed an HC group [16].

The neuropsychological performance profile obtained in our study with 428 participants showed a gradation in the expected direction: ICU-PCC < H-PCC < M-PCC < HC. After controlling for the variables that differed between groups, we found significant differences for the six neuropsychological tests. Post-hoc group comparisons showed that the significant differences arose mainly from the contrast between the HC and ICU-PCC groups. These tests measured global cognition (MoCA), executive functions-mental processing speed (Digit symbol, TMT-B, Phonetic Fluency), and social cognition (RMET). Additionally, the TMT-B test distinguished between ICU-PCC and M-PCC participants.

Our findings partially agreed with those of a study with 213 participants and a similar design to ours [16]. In that study, the severity of COVID-19 was related to deterioration in an overall cognitive score and the attention domain. Some of the tests used to define the attentional domain in that study were also used in our study (Digit symbol, Stroop), while one test that was not used in the present study (Continuous Performance Test) was more sensitive to attention. Although depression and post-traumatic stress disorder were controlled in their overall score analysis, they were not controlled in the attention analysis. The authors of that study reported a relationship between executive function impairment and severity, but this relationship was observed only in men. In our sample, this relationship appeared regardless of sex. Our results referring to the relationship between executive function impairment and the severity of COVID-19 also agree with those of another study [13]. However, that study did not distinguish between hospitalized and ICU participants. The hospitalized patients in our sample did not differ from the outpatients in any test. In contrast, the ICU patients differed from the outpatients in two measures.

Although the neuropsychological profile indicates impairment in the executive domain, tests grouped under executive functions can also be considered to involve processing speed. Several previous studies have related slowness with illness severity [5, 11, 15]. Our results support this relationship. Long-term slower mental speed processing has been linked to hypoxemia in individuals with acute respiratory distress syndrome (ARDS) [48]. Silent hypoxemia is a common feature in SARS-CoV-2 infections [49]. This trait caused delays in patient treatment, particularly during the first wave of the pandemic, which worsened the patients’ prognosis [50]. The integrity of white matter across the brain is related to processing speed and, more generally, to intellectual ability [51, 52]. White matter intensities have been shown to be associated with nocturnal hypoxemia [53] and hypoxic-ischemic brain injury in COVID-19-related ARDS [54]. Consistent with these findings, effects on the white matter have been reported to occur a year after COVID-19, specifically in the corona radiata, corpus callosum, and superior longitudinal fasciculus, particularly in post-ICU individuals [55]. COVID-19-induced white matter injury may be mediated by hypoxia as well as indirect viral invasion [56, 57], the systemic inflammatory response [58], or coagulopathy [59].

COVID-19 severity was not related to memory in our research, even though this relationship has been reported previously [12–14]. This result was unexpected due to the poor memory performance in the entire sample of PCC individuals in comparison with the HC group in our previous study [7]. The high prevalence of depression and anxiety symptoms and fatigue in our groups may explain this finding. In our previous study, fatigue, depression, and anxiety symptoms explained part of the memory performance variance in our PCC groups. Here, when we analyzed the data without controlling for emotional variables and fatigue, the H-PCC and M-PCC participants' learning was inferior to that in the HC group. In addition, the M-PCC group demonstrated poorer long-term memory and recognition than the HC group. Numerous studies have found a link between depression and memory problems in post-COVID individuals [60–62]. The causal connection between depression and memory impairment is, however, uncertain.

In contrast to the findings reported in other studies [10, 63], we did not find differences in cognitive impairment between M-PCC and HC participants. The previous studies performed cognitive assessments of participants 3–6 months after the positive COVID-19 test. In contrast, cognitive assessments for the M-PCC group in the present study were performed an average of eleven months from the acute infection, when most participants may have recovered, at least in part. Most post-COVID symptoms decrease between 3 and 12 months [64], and this change has also been reported in the cognitive symptoms [12]. One study showed no differences between patients with mild- moderate COVID-19 and HCs 4 months post-infection. However, the groups in that study showed remarkable differences in anxiety, depression, and stress [62]. On the other hand, one study evaluating mild COVID-19 individuals at 11 months found several impaired cognitive measures relative to HC. Nevertheless, these authors did not assess whether their participants had fatigue or mood disturbances [65].

Different pathophysiological pathways for brain damage are probably implicated in mild, hospitalized, and critical cases of COVID-19. Despite the possibility of shared pathophysiological mechanisms, assumptions can be made for each group of patients. Mild cases may be caused directly by the virus (olfactory channel of entry) [66, 67]. The degree of systemic inflammation and level of hypoxemia are presumably higher in moderate-COVID-19 individuals [68]. In addition to more severe hypoxemia, systemic inflammation, and organ failure, brain injury may result from ICU therapies, including bed rest, life support equipment, and drugs in critical patients [69].

As a second aim, we investigated the relationship between acute symptoms and long-term cognitive outcomes. We identified five acute symptom components and found correlations between some of these components and long-term cognitive performance. “Neurologic/Pain/Dermatologic,” “Digestive/Headache, Respiratory/Fever/Fatigue/Psychiatric,” and “Smell/Taste” predicted 28% of the variance in global cognition. The “Neurologic/Pain/Dermatologic” component also explained 12% of the variance in attention and WM, and the “Neurologic/Pain/Dermatologic” and “Respiratory/Fever/Fatigue/Psychiatric” components together explained 23% of variance in verbal memory. Finally, 24% of the variance in executive function was accounted for the “Neurologic/Pain/Dermatologic,” “Respiratory/Fever/Fatigue/Psychiatric,” and “Digestive/Headache” components. These three components included the symptoms limb weakness, paresthesia, muscle and joint pain, respiratory issues, fever, depression, anxiety, psychotic symptoms, fatigue, dizziness, and headache.

These results may provide insights into the mechanisms underlying cognitive changes. The fact that the initial symptoms explain some of the variations in long-term cognition suggests that the brain regions responsible for these cognitive tasks were affected, and some of this impairment may have occurred during the acute phase of the illness. The Neurological/Pain/Dermatological, Respiratory/Fever/Fatigue/Psychiatric and Digestive/Headache components included symptoms that develop during systemic inflammation (pain, fatigue, fever, limb weakness, and paresthesia) and neuroinflammation (headache, dizziness, limb weakness, paresthesia, and mood alterations), although these components cannot be explained in terms of inflammation.

We speculate that long-term cognitive impairment could have been caused by sustained systemic or neurological inflammation. Infections result in systemic inflammation and are associated with activation of microglial cells and the appearance of cognitive deficits. Neuroinflammation is caused by activation of microglial cells and the overexpression of proinflammatory cytokines, both of which are induced by the peripheral immune system [70].

In the initial phase of the study, critical patients showed impairment in global cognition, executive function, and social cognition. The variance of these cognitive areas is partially explained here by acute symptom variables. In addition to the inflammation mechanisms underlying the Neurologic/Pain/Dermatologic and Respiratory/Fever/Fatigue/Psychiatric factors, the added Digestive/Headache factor provides an alternative pathophysiological mechanism to explain executive function impairment. The hypothalamus regulates symptoms such as nausea and loss of appetite. SARS-CoV-2 has been suggested to use the nervus terminalis rather than the olfactory nerve as a direct pathway to infect the brain from the nasal cavity [71]. Bypassing the olfactory bulb, nerve terminal neurons project straight to locations in the brain, including the hypothalamus. Infection of the hypothalamus can produce these symptoms and allow the infection to spread to the medial prefrontal lobe [72], contributing to the pathophysiology of executive dysfunction.

Although the results of the verbal memory, attention, and working memory tests did not differ significantly between groups in the initial phase of the study, models predicting the early symptomatology were identified for the components corresponding to these tests. However, language impairments were not predicted by any symptom factor. Instead, these impairments were predicted by demographic variables. Since emotion recognition is associated with the orbitofrontal cortex and temporal regions, we anticipated that the route of entry of the virus through the olfactory system could cause damage to these structures. However, patients with milder disease were more likely to experience impairments in smell and taste, whereas the ICU group, with the most severe condition, demonstrated the poorest social cognition. In this model, obesity, a chronic inflammatory condition [73] linked to the severity of COVID-19 [74], served as an explanatory variable. In addition to the risk posed by chronic inflammation, severely obese patients show considerable management issues in the ICU, particularly for the respiratory level [75]. Therefore, the impairment of the brain structures responsible for recognizing emotions should be attributed to indirect mechanisms, such as hematogenous pathways of virus entry to the central nervous system or systemic inflammatory mechanisms, and not to the direct action of the virus. We cannot rule out the possibility that sedated and intubated participants’ self-reported baseline symptoms were not as accurate as those with less severe COVID-19. Thus, we may have lacked complete and reliable data regarding symptoms such as anosmia/ageusia in severely ill patients.

The limitations and strengths of the study require consideration while interpreting the findings. A major limitation refers to the collection of initial symptoms, which were self-reported through a questionnaire in the first session with the patient; the questionnaire itself was based on the symptoms most frequently reported in the literature. Thus, the presence of initial symptoms was recorded and scored retrospectively, which may have introduced recall bias. Moreover, we collected data for the presence and duration but not the intensity of each symptom. We did not use objective severity measures such as hypoxemia, days of sedation or weaning, or blood inflammatory levels, and the analysis was based solely on the reported symptoms. Since these factors may better explain the cognitive deficit, these variables will be examined in depth in future studies to understand the pathogenesis of cognitive dysfunction in PCC individuals.

On the other hand, our sample size was reasonably large and represented the full spectrum of COVID-19 severity. Although the control group was not optimal because we had to control for some variables statistically, it was tested simultaneously with the COVID-19 participants, with the HCs experiencing the same pandemic circumstances. Unlike other studies, our participants were selected on the basis of inclusion criteria that precluded the presence of neurological, psychiatric, or systemic illnesses before COVID-19, conditions that could have influenced the cognitive findings. In addition, the cognitive examination was carried out in person with an extensive neuropsychological battery commonly used in the clinical context, which validated its applicability.

In conclusion, the results of this study showed evident long-term impairments in patients with severe COVID-19 requiring ICU admission, although hospitalization per se did not involve long-term neuropsychological sequelae. Global cognition, executive function, and social cognition were the domains most affected by the severity of COVID-19. For the initial symptomatology, the factors Neurologic/Pain/Dermatologic, Respiratory/Fever/Fatigue/Psychiatric, and Digestive/Headache explained part of the variance of global cognition, attention and working memory, verbal memory and executive function.

## Supplementary Information

Below is the link to the electronic supplementary material.

Supplementary material 1 (DOCX 386 KB)

## Data Availability

The raw data supporting the conclusions of this article will be made available by the authors, without undue reservation.
