# Supplementary material for: COVID-19 severity is related to poor executive function in people with post-COVID conditions
Source: J Neurol. 2023 Mar 20;270(5):2392–408. doi: 10.1007/s00415-023-11587-4 (PMC10026205; doi:10.1007/s00415-023-11587-4)
Supplement: Supplementary file 1 — Supplementary material 1 (DOCX 386 KB) [file 415_2023_11587_MOESM1_ESM.docx]

**Supplementary table 1. Means and standard errors for the neuropsychological variables for the PCC severity and HC groups adjusted by age, sex, educational level, estimated IQ***

|  | **ICU-PCC**  **(n=77)** | **H-PCC**  **(n=73)** | **M-PCC**  **(n=169)** | **HC**  **(n=109)** |  |  |  |  |  |
| --- | --- | --- | --- | --- | --- | --- | --- | --- | --- |
|  | **M_adj_ (SE)** | **M_adj_**  **(SE)** | **M_adj_**  **(SE)** | **M_adj_**  **(SE)** | **F** | **p** | **η_p_²** | **Post-hoc**  **Bonferroni** | **p** |
| MoCA | 25.91  (0.30) | 26.02  (0.30) | 26.07  (0.19) | 27.55  (0.25) | 9.418 | .0001 | .065 | ICU < HC  H < HC  M < HC | .0001  .0001  .0001 |
| RAVLT Sum | 45.63  (0.98) | 43.71  (1.00) | 43.76  (0.66) | 48.02  (0.88) | 2.366 | .071 | .018 | H < HC  M < HC | .008  .0001 |
| RAVLT Immediate recall | 8.77  (0.32) | 8.96  (0.33) | 8.85  (0.21) | 9.58  (0.27) | 1.866 | .135 | .013 |  |  |
| RAVLT Delayed recall | 8.70  (0.36) | 8.77  (0.36) | 8.75  (0.23) | 9.84  (0.30) | 3.320 | .020 | .024 | M < HC | .023 |
| RAVLT Recognition | 12.42  (0.28) | 12.09  (0.28) | 12.08  (0.18) | 12.89  (0.23) | 2.941 | .033 | .021 | M < HC | .032 |
| Digit Span  Forward | 5.38  (0.14) | 5.57  (0.14) | 5.71  (0.09) | 5.56  (0.12) | 2.927 | .034 | .021 | ICU < HC | .036 |
| Digit Span  Backward | 4.30  (0.14) | 4.38  (0.14) | 4.47  (0.09) | 4.67  (0.12) | 1.515 | .210 | .011 |  |  |
| Digit Symbol | 62.12  (1.96) | 62.06  (2.00) | 65.69  (1.28) | 74.00  (1.63) | 9.701 | .0001 | .066 | ICU < HC  H < HC  M < HC | .0001  .0001  .0001 |
| TMT-A (time) | 43.59  (2.42) | 37.21  (2.47) | 36.44  (1.59) | 32.66  (2.02) | 3.803 | .010 | .027 | ICU > HC | .005 |
| TMT-B (time) | 110.51  (6.48) | 87.98  (6.67) | 80.42  (4.20) | 70.61  (5.34) | 7.406 | .0001 | .052 | ICU > HC  ICU > M | .0001  .0001 |
| TMT-B-A (time) | 68.58  (4.95) | 51.02  (5.07) | 43.85  (3.22) | 37.97  (4.08) | 7.757 | .0001 | .054 | ICU > HC  ICU > M | .0001  .0001 |
| Stroop Words | 92.53  (2.51) | 93.24  (2.53) | 92.90  (1.621) | 100.60  (2.08) | 3.389 | .018 | .024 | M < HC | .020 |
| Stroop Colors | 64.64  (1.61) | 63.67  (1.04) | 63.75  (1.04) | 70.35  (1.33) | 5.826 | .0001 | .041 | H < HC  M < HC | .012  .0001 |
| Stroop Interference | 38.35  (1.21) | 37.54  (1.22) | 38.44  (0.78) | 43.85  (1.00) | 7.790 | .0001 | .054 | ICU < HC  H < HC  M < HC | .005  .0001  .0001 |
| Phonetic fluency (PMR) | 39.33  (1.35) | 42.43  (1.39) | 42.11  (0.89) | 47.12  (1.14) | 6.923 | .0001 | .048 | ICU < HC  M < HC | .0001  .003 |
| Semantic fluency (animals) | 20.68  (0.61) | 20.61  (0.63) | 21.10  (0.40) | 23.30  (0.51) | 5.446 | .0001 | .038 | ICU < HC  H < HC  M < HC | .009  .008  .004 |
| BNT | 51.35  (0.56) | 51.52  (0.57) | 52.60  (0.37) | 52.94  (0.47) | 2.109 | .098 | .015 |  |  |
| RMET | 21.24  (0.43) | 22.31  (0.43) | 22.65  (0.28) | 23.54  (0.36) | 5.362 | .0001 | .037 | ICU < HC  ICU < M | .0001  .043 |

PCC = post-COVID condition; ICU = Intensive care unit; H = Hospitalized; M = mild; HC = healthy control; MoCA = Montreal Cognitive Assessment; RAVLT = Rey’s Auditory Verbal Learning Test; TMT = Trail Making Test; BNT=Boston Naming Test; RMET= Reading the Mind in the Eyes Test.

^*^ By means of Word Accentuation Test; η_p_² effect size is as follows: η_p_^2^ = .009, small; η_p_^2^ = .059, medium; η_p_^2^ = .139, large.

**Supplementary table 2. Initial symptom factors for the PCC severity groups**

|  | **ICU**  **(n=77)** | **H**  **(n=73)** | **M**  **(n=169)** |  |  |  |  |  |
| --- | --- | --- | --- | --- | --- | --- | --- | --- |
|  | **M_adj_**  **(SE)** | **M_adj_ (SE)** | **M_adj_ (SE)** | **F** | **p** | **η²** | **Post-hoc test** | **p** |
| Digestive/Headache | -0.37  (0.81) | 0.08  (0.89) | 0.21  (0.21) | 9.780 | .0001 | .059 | M > ICU | 0.001 |
| Respiratory/Fever/Fatigue/Psychiatric | 0.24  (1.16) | 0.15  (0.89) | -0.18 (0.93) | 6.110 | .002 | .038 | ICU > M  H > M | .006  .045 |
| Neurologic/Pain/Dermatologic | -0.01  (0.91) | -0.19  (0.88) | 0.09  (1.07) | 1.946 | .145 | .000 |  |  |
| Smell/Taste | -0.38  (0.95) | -0.33  (0.89) | 0.32  (0.96) | 20.604 | .0001 | .117 | M > ICU  M > H | 0.001  0.001 |
| Cold | -0.12  (0.99) | 0.14  (0.97) | -0.00  (1.01) | 1.398 | .249 | .009 |  |  |

PCC = post-COVID condition; ICU = Intensive Care Unit; H = hospitalized; M = mild; HC = healthy control

η_p_² effect size is as follows: η_p_^2^ = .009, small; η_p_^2^ = .059, medium; η_p_^2^ = .139, large.
